# Supplementary figures and images for: Efficacy and safety of berberine plus 5-ASA for ulcerative colitis: A systematic review and meta-analysis
Source: PLoS One. 2024 Sep 6;19(9):e0309144. doi: 10.1371/journal.pone.0309144 (PMC11379390; doi:10.1371/journal.pone.0309144)

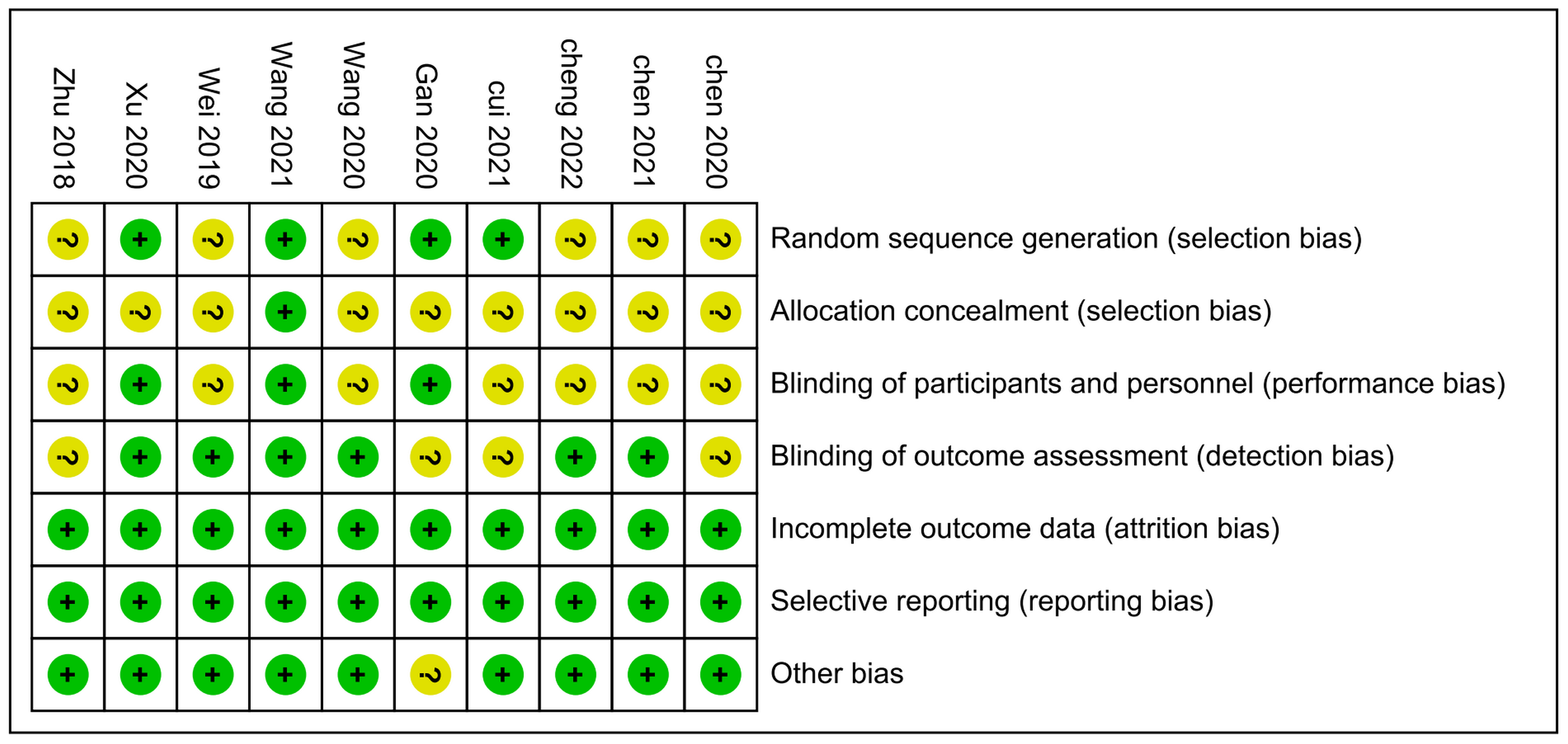

Supplement: S1 Fig — (TIF) [file pone.0309144.s004.tif]
