# Supplementary material for: Efficacy and safety of berberine plus 5-ASA for ulcerative colitis: A systematic review and meta-analysis
Source: PLoS One. 2024 Sep 6;19(9):e0309144. doi: 10.1371/journal.pone.0309144 (PMC11379390; doi:10.1371/journal.pone.0309144)
Supplement: Attachment — Submitted filename: PLOS One Review_ BBR and UC.docx [file pone.0309144.s005.docx]

Overall, I think this analysis is of interest to the field and a good contribution to the literature. However, there are a few overarching concerns (“General Comments”) that must be addressed before I can recommend for the publication, in addition to a detailed breakdown of items associated with each section of the manuscript (“Detailed Comments”).

**GENERAL COMMENTS**

1. There are many locations where the authors overstate the findings/context of the study to implicate “BBR as a therapy for UC” when the study actually evaluates the efficacy of “BBR as a complementary therapy to support/enhance standard treatments.”
   a. Because BBR is being used in addition to standard treatment in all studies and is not being used alone in comparison to standard treatment, you are not evaluating BBR as an alternative therapy. So, in locations where it is implied the study is evaluating BBR in "treating disease,” it should be reworded to something like, "supporting disease management".
   b. The locations of these overstatements are highlighted in detail in “Detailed Comments.”
2. Many issues with citations: There is a considerable lack of citations for physiological and mechanistic statements throughout the article. These are highlighted in detail in “Detailed Comments.” Also, two of the ten articles included in the analysis are not properly cited in the References (in-text Figures include different author names for references 22 and 24)
3. Need additional details in Methods for reproducibility (These are highlighted in detail in “Detailed Comments.”), especially around data extraction.
4. The lack of availability of many of these articles in the English language and on many databases common to Europe and the Americas presents a major barrier to researchers outside of China/Asia who may want to follow up on these articles. I unsuccessfully tried to search for many of these articles through Medline, EMBASE, PubMed, Google Scholar, and three separate university libraries for institutions that I am affiliated with (one of them being a major medical school library)—so, I was unable to confirm many methods or double-check measures. Because of this, **authors must include which database they accessed each article** from and any identifiable database numbers (e.g., DOI) in Table 1 to facilitate the reproducibility of the search and access to these papers.

**DETAILED COMMENTS**

I. **Abstract/Introduction**
   1. Line 31: This is misleading. Please re-word to, "...that administer BBR **in conjunction with standard therapy** for to support the treatment of UC".
   2. Line 63: keep style of ratio reporting the same...E.g., you transition from "286.3 per 100,000" to "11.3/100,000"
   3. Line 74: Italicized genus and species names.
   4. You must place your current review/meta-analysis in the context of any existing systematic reviews and meta-analysis on similar content (which do exist)—Describe what content those reviews/meta-analysis include and how your meta-analysis is different and contributes to the literature.
   5. Lines 82-84 “The Purpose”: This is misleading; please clarify the purpose to reflect what the meta-analysis is truly evaluating, which is the efficacy of BBR as a complementary therapy to support treatment.
II. **Methods**
    1. Search strategy:
       i. Just semantics here…but PubMed is technically not a “database”, it is a platform for the database Medline.
       ii. Were there any limits or filters used?
       iii. Were there any programs used to convert search syntax across databases (for example, Polyglot)?
    2. Inclusion criteria: We need more clarity here
       i. The second sentence of this section begins “The exclusion criteria…” Although, this section is supposed to be about inclusion criteria. Reading this section makes it seem as if you excluded RCTs and studies where BBR was used in conjunction with a certain standard therapy – however, I believe you mean for these to be inclusion criteria.
    3. Exclusion Criteria: We need more information here for reproducibility.
       i. “Statistical inaccuracies”: Elaborate and define what you consider inaccuracies, and include how these inaccuracies were identified.
       ii. “Inadequate original data”: Elaborate and define what you consider inadequate original data, and include how this was identified.
    4. Secondary outcomes: We need more clarity and additional information here
       i. There is a lot of information in the Results section for each measure that actually belongs here, in the methods.
          1. This section would benefit from more clearly defining each measure, what it is used for, and why. Perhaps consider breaking this down into the “three domains” you highlight in the discussion?
          2. Once the reader gets to the discussion, it becomes clear that not all articles contain all measures. In the methods, please define the number of articles used for each outcome.
    5. Literature screening, data extraction, and quality assessment: We need much more information here for reproducibility.
       i. Were there any automation tools or other programs used in the extraction and screening process?
       ii. Were any extraction forms used pretested? (for example, if you created or used an Excel template, were these pretested?)
       iii. Was extraction conducted with standardized instructions?
       iv. Were there specific domains for extraction used? If so, what were they?
       v. What instruments did you use for risk of bias assessment (Example: version 2.0 of the Cochrane Risk-of-Bias (RoB) instrument)?
       vi. How did you handle missing data?
III. **Results**
     1. Clinical efficacy rate:
        i. Lines 204-205: This is the first place where is becomes clear that you are investigating the efficacy of BBR as a complementary therapy, not as a therapy on its own. This needs to be clarified in the introduction and “purpose” of the study.
     2. Baron Score:
        i. Lines 208-213: this definition belongs in the Methods section
     3. DAI score:
        i. Lines 221-224: this definition belongs in the Methods section
     4. Symptom amelioration analysis:
        i. Lines 231-233: This information does not belong in the results section. It belongs in either the introduction *or* the methods section when you introduce/define the biomarkers being measured.
     5. Inflammatory biomarkers:
        i. Lines 246-250: This information does not belong in the results section. It belongs in either the introduction *or* the methods section when you introduce/define the biomarkers being measured.
     6. Immune Response
        i. Lines 270-721: regarding the CD4+:CD8+ T cell ratio as a gauge of immune function; this must be cited with a reference.
        ii. Line 273: “Increase in CD4+ T cells”
            1. I cannot access the articles cited, so I cannot check this: Did these studies specify which CD4+ T cell compartment they measured? Is these T effector cells only, or all CD4+ T cells which include T regulatory cells? This is important to further contextualize the physiological relevance of this finding. CD4+ T cells encompass both T effector cells (e.g., Th1, Th2, Th17, et) AND T regulatory cells, which are immunosuppressive.
IV. **Discussion**
1. General:
   a. It is a little unclear throughout the Discussion which statements are regarding results from your meta-analysis and which are from other studies.
   b. MANY studies use BBR in rodent models of UC that shed light on possible mechanisms of action; It would be useful to place some of your findings in the context of these mechanisms. You *very* lightly touch on the immunological mechanism, but this can be discussed in more detail.
   c. BBR also clearly interacts with the gut microbiome to modulate immune function and intestinal health. However, this important interaction is not mentioned at all.
   d. Lines 316-317: “three domains…”
      i. Breaking down your outcomes into these “three domains” is a great way to organize the information for the reader. Perhaps consider introducing “domains” of outcomes earlier on in the manuscript.
   e. Line 320: “casting doubt on robustness..”
      i. I would steer away from the term "casting doubt" and focus more on facts... limited sample sizes can make studies underpowered and, if homogeneous samples were used (i.e., geographically, or demographically), results may not be generalized to the wider population.
   f. Lines 331-334: this sentence is a little hard to follow
   g. Line 335: “surge in patients Baron scores”
      i. In the following sentence, you mention BBR *lowering* Baron scores and that lowering of score is correlated to symptom relief. However, here in line 335 you mention "surge" in Baron score, which indicates an elevation in score. These two statements are contradictory.
2. Limitations: There are limitations to this study which must be highlighted in the discussion
   a. All articles included in the meta-analysis are from China/Asia. This limits the generalizability of the results.
   b. Table 1 highlights differences in administration route and treatment time. Could these factors be confounders to analysis?
3. Overstating findings: You are not evaluating the efficacy of BBR as an alternative therapy in this meta-analysis, you are evaluating BBR as a **complementary** therapy. Thus, your meta-analysis supports the concept of “BBR as a safe and efficacious *complementary* intervention to support disease management”. Specific lines in manuscript that overstate findings/context of article:
   a. Line 309-310: “BBR has emerged as a promising alternative therapy for treating this disease”
   b. Line 312: “…application of BBR in treating UC…”
   c. Line 371: “BBR is a safe and efficacious intervention”
4. Discussion of immune responses:
   a. Lines 340-342: regarding IL-8 secretion; this must be cited with a reference
   b. Lines 342-344: Regarding BBR effect on NFkB and cytokines; this must be cited with a reference
   c. Lines 351-352: The wording of this is not entirely correct and is a little misleading. An inflammatory insult does not cause de novo CD4+ and CD8+ cell "synthesis" (any "synthesis" would happen in the thymus)... I think what you are trying to convey here is that naive CD4+ T cells and naive CD8+ T cells become activated and differentiate into effector cells, memory cells, etc. Regarding the use of the word "evolution"… you should really be referring to this as the “activation and differentiation" of naive CD4+ T cells. Please refer to the literature on naive CD4+ T cell activation and differentiation into effector cell phenotypes, memory cell phenotypes, and regulatory cell phenotypes to improve the accuracy of these statements.
   d. Lines 354-355: Information cited with Reference 35; this is not the best reference for this statement. Please double-check.
   e. Lines 355-357: Regarding the ratio of CD4+:CD8+ T cells and which ratio is indicative of immune competency; this must be cited with a reference.
      i. Also, it is a little unclear what the “range” of 1 to 2 means. Is this the range of ratios?
   f. Line 358: “…enhancing immunological functions.”
      i. This statement is very general, and its meaning is unclear. Please clarify what you mean by "enhancing." Is the immunological tone of intestinal mucosa becoming more activated and inflammatory? Is it becoming more suppressed?
V. **Other:**

- Concerns over citations: Incorrect References section citations of two of the ten articles included in the meta-analysis
- Citation 22: *Pengli W. Salt of berberine combined with mesalazine for the treatment of ulcerative colitis: an observational study on the therapeutic effects[J]. Clinical Medical Engineering, 2020,27(6):771-772.*
  a. Issue 1: Is this the “Wang 2020” article? If so, please correct the citation.
  b. Issue 2: There is concern over the title which mentions, “observational study” where the studies included in the meta-analysis should be randomized controlled trials. However, I cannot find this study through searches of Medline, Embase, or my institutional libraries to check this study. Is it possible to link these studies in the references section to DOIs or some other database identifier number for easy access?
- Citation 24: *Liuliu W. The therapeutic efficacy of berberine combined with mesalazine and its impact on inflammatory factors in patients with distal ulcerative colitis[J]. Proceeding of Clinical Medicine, 2019,28(7):506-508.*
  a. Issue 1: Is this the “Wei 2019” article? If so, please correct the citation.
- Concerns over citations: Lack of in-text citation for many statements
  - Articles listed in Table 2 must be cited with the citation number of the article (as numbered in the references section) so the full citations can be easily found.
  - Line 80 “Xu” article: check in-text citation style. Should a date be included?
  - Lines 269-271: This sentence regarding the ratio of CD4+:CD8+ being used as an indicator of immune function must be cited with a reference.
  - Lines 308-309: This sentence must be cited with a reference.
  - Lines 322-325: Cite which references this applies to.
  - Lines 326-327: The sentence regarding remission and relapse must be cited with a reference.
  - Lines 329-330: The sentence regarding endoscopic assessments must be cited with a reference. It is also unclear if this is from your meta-analysis or a different study.
  - Lines 354-355: Information cited with Reference 35; this is not the best reference for this statement. Please double-check.
  - Lines 355-357: Regarding the ratio of CD4+:CD8+ T cells and which ratio is indicative of immune competency; this must be cited with a reference.
  - Lines 342-344: Regarding BBR effect on NFkB and cytokines; this must be cited with a reference
  - Lines 340-342: regarding IL-8 secretion; this must be cited with a reference
