# Supplementary material for: Efficacy and safety of berberine plus 5-ASA for ulcerative colitis: A systematic review and meta-analysis
Source: PLoS One. 2024 Sep 6;19(9):e0309144. doi: 10.1371/journal.pone.0309144 (PMC11379390; doi:10.1371/journal.pone.0309144)
Supplement: Attachment — Submitted filename: Responses to Reviewers.docx [file pone.0309144.s006.docx]

Dear Editors and Reviewers:

We gratefully appreciate the editors and all reviewers for their time spent making positive and constructive comments. These comments are all valuable and helpful for revising and improving our manuscript entitled “Efficacy and safety of berberine plus 5-ASA for ulcerative colitis: A systematic review and meta-analysis” (PONE-D-24-02154).

We have made our utmost efforts to improve the manuscript in the following ways: (1) avoiding overestimating the effects of berberine and accurately describing its effects; (2) adding necessary citations; (3) supplementing explanations or rewriting parts that are difficult to understand. These revisions will help enhance the overall quality of the manuscript. The reviewer comments are laid out below in italicized font and specific concerns have been numbered. We have studied comments carefully and have made corrections which we hope meet with approval. Our response is given in normal font and changes/additions to the manuscript are given in red.

Thank you and best regards.

Yours sincerely,

Lili Yang

Kaifeng Central Hospital

**Responses to Reviewers**

**Editor Comments**

***Comment 1****: Some of the subject studies are not in English is a barrier to many other researchers.*

**Response 1:** Thank you for your comment. We were sorry for our careless mistakes. We have already changed the non-English text below Table 1 to English text. (line 188-190 on page 10).

***Comment 2:*** *overstatement of claims about use context of berberine*

**Response 2:** We would like to express our sincere gratitude for your professional review of our article. Your feedback and suggestions were very helpful and insightful. You are such a patient and responsible reviewer, and I feel very honored to have your assistance. Based on your suggestion, we have made revisions. When describing the effects of berberine, we have rephrased our wording to avoid overstating the effects of berberine. For example, we have used phrases like "BBR as a safe and efficacious complementary intervention to support disease management", "BBR in conjunction with standard therapy to support the treatment of UC", "Berberine plus 5-ASA for ulcerative colitis ", "BBR demonstrates substantial efficacy in treating UC without causing severe adverse reactions and may serve as a viable complementary therapy” to accurately express the effects of berberine.

(line 1 on page 1), (line 41-42 on page 2), (line 247 on page 12), (line 409-410 on page 18) et al.

***Comment 3:*** *Regarding the section on adverse events, did any of the studies report glycemic control indicators (glucose, insulin, HOMA-IR, etc.) or changes in body mass/weight?*

**Response 3:** Thanks for your comment. We have carefully read the literature included this time, however, we did not find any studies that reported these indicators. The issue you mentioned is interesting, and we will focus on it in our future research.

***Comment 4:*** *I think that you need to carefully re-visit the T cell section. First, the claim that BBR “augmented” CD8+ is not supported by simple visual inspection of the data; further even if some statistical test alleges significant difference here, it is not supported by the actual numbers/biology whose differences are not truly substantive.*

**Response 4** Thank you for your professional and meticulous review; we apologize for our oversight. The expression "augmented CD8+" was incorrect. After reevaluating, we have revised the sentence in the manuscript from " The data indicated that BBR significantly augmented CD8+ T cells (*P* < 0.00001)" to "The data indicated that combining BBR with 5-ASA does not significantly alter the expression of CD8+ T cells compared to using 5-ASA alone." We thank you again for your expert guidance. (line 281-283 on page 14).

***Comment 5:*** *Second, CD4+ changes could include changes in T regulatory (Treg) cell populations. Did any studies report FOXP3 and/or CD25 expression? This should be examined and discussed, even if none of the reported studies indicated such. These are important cells to consider in pathologies such as UC.*

**Response 5:** Thank you for your professional review. While the original study did not report on the expression of FOXP3 and/or CD25, these elements are crucial. Following your advice, we have added related discussions in the manuscript. The specific additions are as follows: "Regrettably, the study did not report key immunological factors, FOXP3 and CD25. FOXP3, serving as the principal marker for Tregs, is critical for their suppressive function^[1]^. Research by Zhang et al. suggests that diminished expression of FOXP3 in the intestinal tissues of UC patients may lead to an imbalance in immune regulation, exacerbating the pathological conditions and inflammatory responses^[2]^. Similarly, reduced expression of CD25 can weaken the inhibitory capacity of Tregs, aggravating the pathology and inflammation associated with UC[3]." (line 391-398 on page 18).

***Comment 6:*** *Finally, what does increased CD4+ mean in the context of “favours control” as depicted in the figure?*

**Response 6:** Thank you for your guidance. We have not expressed ourselves before, but with your help, we can now articulate more precisely, as follows: "The increase in CD4+ T cells may promote an increase in the number of regulatory T cells, which helps alleviate the inflammatory response in UC." Additionally, we have made modifications to the manuscript accordingly. (line 278-280 on page 13 ).

***Comment 7:****The statements and the figure do not seem to be in agreement.*

**Response 7:**Thank you for your comment. We are sorry for our carelessness. We have rechecked and made specific modifications as follows: Firstly, change (I^2^ = 81%, *P* < 0.00001) into (I^2^ = 81%, P = 0.02). Change (P = 0.0004) into (P = 0.0001). Additionally, we have changed “The data indicated data that BBR significantly augmented CD8+ T cells (*P* < 0.00001).” to “The data showed that combining BBR with 5-ASA does not significantly alter the expression of CD8+ T cells compared to using 5-ASA alone(P = 0.50).” (line 282-284 on page 14).

***Comment 8:****Without further details as to the phenotypes of CD4+ T cells, one cannot really say much about favoring one group vs. another; for example, an increase in Th17 cells could actually indicate worsening of cellular pathology, whereas an increase in Treg may indicate amelioration.*

**Response 8:**Thank you for your detailed comments and professional guidance. As you pointed out, CD4+ T cells include both T effector cells (such as Th1, Th2, Th17, etc.) and T regulatory cells, which are immunosuppressive. They have different physiological functions; for instance, an increase in Th17 cells may indicate the progression of UC, while an increase in Treg cells may suggest a mitigation of UC. We revisited the article and realized that the mentioned CD4+ T cell compartment includes both T effector cells and T regulatory cells. At the same time, we also provide the DOI or link of the articles in the references for easy access by the readers.

***Comment 9:*** *Lines 148-152 need to be re-written to indicate the subject studies used these techniques. As currently written the statements could be construed that you conducted these experiments.*

**Response 9:**Thank you for your reminder; your guidance has been immensely helpful. You also mentioned in the following comment that this section was overly detailed and suggested we streamline it. The revised content is as follows:

(4) Inflammatory cytokines: IL-6, IL-8, TNF-α, IL-10. Levels of IL-6, IL-8, and TNF-α decrease, while IL-10 increases, indicating a reduction in the inflammatory response of UC.

(5) Immune response: CD4+ T cells, CD8+ T cells, CD4+/CD8+ ratio. CD4+ and CD8+ T lymphocytes are distinct subpopulations that play pivotal roles in the immune response, with the CD4+/CD8+ ratio serving as a gauge of immune function in UC patients. (6) Adverse reaction.(line 139-146 on page 6).

***Comment 10*** *Table 1 descriptors need to be re-written in English.*

**Response 10**hank you for your comment. We were sorry for our careless mistakes. We have already changed the non-English text below Table 1 to English text. (line 188-190 on page 10).

***Comment 11*** *I suggest combining Figures 3, 4, and 5 into a single figure.*

**Response 11** Thank you for your suggestion. Figures 3, 4, and 5 represent the clinical efficacy rate, Baron score, and DAI score, respectively. Each represents different aspects, hence separating them into individual charts will make the results clearer. Therefore, we have decided not to combine them, but we equally appreciate your valuable suggestion.

***Comment 12*** *Lines 256-257: more should be stated about the IL-10 results, particularly discordance between control and BBR intervention.*

**Response 12**Thank you for your guidance, we have added the following content.

IL-10 is a common anti-inflammatory factor, and the higher its expression level, the milder the inflammatory response. 3 studies suggest that compared to using 5-ASA alone, the combination of BBR and 5-ASA can increase the expression of IL-10, helping to alleviate the inflammatory response in patients with UC. (line 261-264 on page 13).

***Comment 13*** *Delete the first sentence of line 340. It is unnecessary.*

**Response 13 Thank you for your professional guidance, we have already removed that sentence.**

***Comment 14*** *There are currently no detailed figure legends. This should be addressed.*

**Response 14**Thank you for the reminder. We have already placed each figure legend directly after the paragraph in which they are first mentioned.

**Journal Requirements:**

***Comment 15*** *Please ensure that your manuscript meets PLOS ONE's style requirements, including those for file naming.*

**Response 15** Thank you for your guidance; we have revised the style of the manuscript according to the requirements, including those for file naming.

***Comment 16****. Please include your tables as part of your main manuscript and remove the individual files. Please note that supplementary tables (should remain/ be uploaded) as separate "supporting information" files.*

**Response 16** Thank you for your meticulous guidance on formatting. We have placed Table 1 in the manuscript as per your instructions. (line 187-190 on page 9-10).

**Reviewer #1:**

**GENERAL COMMENTS**

***Comment 1****: There are many locations where the authors overstate the findings/context of the study to implicate “BBR as a therapy for UC” when the study actually evaluates the efficacy of “BBR as a complementary therapy to support/enhance standard treatments.” a. Because BBR is being used in addition to standard treatment in all studies and is not being used alone in comparison to standard treatment, you are not evaluating BBR as an alternative therapy. So, in locations where it is implied the study is evaluating BBR in "treating disease,” it should be reworded to something like, "supporting disease management".*

**Response 1:** We would like to express our sincere gratitude for your professional review of our article. Your feedback and suggestions were very helpful and insightful. You are such a patient and responsible reviewer, and I feel very honored to have your assistance. Based on your suggestion, we have made revisions. When describing the effects of berberine, we have rephrased our wording to avoid overstating the effects of berberine. For example, we have used phrases like "BBR as a safe and efficacious complementary intervention to support disease management" and "BBR in conjunction with standard therapy to support the treatment of UC" to accurately express the effects of berberine. (line 1 on page 1), (line 41-42 on page 2), (line 247 on page 12), (line 409-410 on page 18)et al.

***Comment 2:*** *Many issues with citations: There is a considerable lack of citations for physiological and mechanistic statements throughout the article. These are highlighted in detail in “Detailed Comments.” Also, two of the ten articles included in the analysis are not properly cited in the References (in-text Figures include different author names for references 22 and 24)*

**Response 2:** Thank you for reading our paper so carefully. We have added citations in the section on physiological and mechanistic statements. Additionally, we have made modifications to the original references 22 and 24 to ensure that the citations are more standardized. After our revisions, the article now cites approximately 20 more references than the previous version. A detailed response will be provided below the comments of the first reviewer.

***Comment 3:*** *Need additional details in Methods for reproducibility (These are highlighted in detail in “Detailed Comments.”), especially around data extraction.*

**Response :** Thank you for your valuable guidance on the manuscript. We have supplemented the methods as requested. The following paragraph is the revised content:

"By screening the titles and abstracts, two authors (Jilei Li and Chenchen Zhang) independently removed duplicate and irrelevant records. Any discrepancies between the researchers were addressed by consulting the third researcher Yanchao Xu. After skimming the full text, articles not meeting the inclusion criteria or meeting the exclusion criteria were discarded. Two independent researchers summarized the extracted literature information into a table following standardized instructions. Data extraction includes the first author, publication year, sample size, age, sex ratio, interventions, treatment method, treatment time, outcome, and source. If the data in the table is missing, it will not be included. The assessment of the quality of the included studies was meticulously conducted following the Cochrane Handbook for Systematic Reviews of Interventions, version 5.4.1, which provided a risk of bias-evaluation tool. We used version 2.0 of the Cochrane Risk-of-Bias (RoB) instrument for risk of bias assessment.” (line 150-157 on page 7).

***Comment 4:*** *The lack of availability of many of these articles in the English language and on many databases common to Europe and the Americas presents a major barrier to researchers outside of China/Asia who may want to follow up on these articles. I unsuccessfully tried to search for many of these articles through Medline, EMBASE, PubMed, Google Scholar, and three separate university libraries for institutions that I am affiliated with (one of them being a major medical school library)—so, I was unable to confirm many methods or double-check measures. Because of this, authors must include which database they accessed each article from and any identifiable database numbers (e.g., DOI) in Table 1 to facilitate the reproducibility of the search and access to these papers.*

**Response 4:** Thank you for your professional guidance. We apologize for any inconvenience our layout may have caused. We also appreciate your suggestions. However, placing the DOI or LINKs in Table 1 would require additional space and could detract from the table's appearance. Therefore, we have uploaded the DOIs or LINKs as an attachment, titled "DOI OR LINK." We have also listed the DOIs or LINKs below for your reference.

1. Chen 2020：DOI: 10.14164/j.cnki.cn11-5581/r.2020.10.063

LINK:<https://kns.cnki.net/kcms2/article/abstract?v=yqeyU9EK6jT70Eh65eKmwK43ydBlFTIDStH0jexxpkVk6vtooH0y2vfgnSk_B8W9hkQO6HVgO9u3iA0BsjexoPSd_J--WD0t21BYlntP4Mda8D3jrgtTQzfcJnacYUHIrh04FGHrhFw=&uniplatform=NZKPT&language=CHS>

2.Chen 2021

LINK:https://kns.cnki.net/kcms2/article/abstract?v=yqeyU9EK6jR_XW5Z3mPDxkyme3NI4DoXhznzuWMqQmDji6RrU67zynGJ97EZJrLDmWYG5B7pv9e6TfxfRDraupGkVHZQ9070plbElSTDFNOcx1vR94oVQkXXzP0jGkDukRYC9DVIErU=&uniplatform=NZKPT&language=CHS

3. Cheng 2022

LINK: <https://kns.cnki.net/kcms2/article/abstract?v=yqeyU9EK6jT1dAmV7rL-KIpLPmpheHbLyT5QSnjXfiXeuX68BMDP33lIhHTW5AoN_Ax8JOa7EnH06ZVvB52FhVxsg2I1_OVecwp7dma3zYV1jEvkS_TugnUCjfAnZR7iTQ1XjkalIyo=&uniplatform=NZKPT&language=CHS>

4. Cui 2021

LINK: <https://kns.cnki.net/kcms2/article/abstract?v=yqeyU9EK6jRX2Bo-wogWiT86QuBWDmzqStLSmOKGvbIBicLxqheF3Vwh3pv3eWHxGpcQA0ZIOwTG506uHe6sFLOF7K7AxeigNHTO8v0W5Qm2bKTFjq33aqLkCgzB9OJvbWfm0gMqUG8=&uniplatform=NZKPT&language=CHS>

5. Gan 2020	doi：10.3969/j.issn.1671-038X.2020.12.10

LINK:<https://kns.cnki.net/kcms2/article/abstract?v=yqeyU9EK6jRcsAtlmqq8ZFMv6NhdyrYXJ2X8pS1HzR0waKf_GqNaKltwpXi1kfNIYPSMlyg5NAq3uwpR-cGMbyHBBZyBf7vIPaQzVFJzZUFluwkNJvRcZ_-krFydhjt1-kx-1LBRLkw=&uniplatform=NZKPT&language=CHS>

6. Wang 2020	doi:：10.3969/j.issn.1674-4659.2020.06.0771

LINK:<https://kns.cnki.net/kcms2/article/abstract?v=yqeyU9EK6jT_Vjj63FIaYSNsre0H_MG8ztgLP_wgjQTv_mrI5vJEw0jvGVfIiBTOSowUqwad8Ze0FhtXnJfVsaNSfzmLSTZjEJdAetXPxIRkpQbtjj98_U837I_XZGBz0hgK2UxGc2w=&uniplatform=NZKPT&flag=copy>

7. Wang 2021	doi：10.3969/j.issn.1004-437X.2021.23.044

LINK:<https://kns.cnki.net/kcms2/article/abstract?v=yqeyU9EK6jQB8ug3mPUBvLR1_WlGczeWrdk5CdCNixabaYYx2-UbRPsV-wbxBqPFr4iPlNlwS02w2xiy3s-KwwHqQTZzX1kEkv241qp3fhzdbPnlhMU_4nb-WJRbaVHuE2ZgxaQERF0=&uniplatform=NZKPT&language=CHS>

8. Wei 2019	doi：10.16047/j.cnki.cn14-1300/r.2019.07.009

LINK:https://kns.cnki.net/kcms2/article/abstract?v=yqeyU9EK6jSOQe4ylN8lXnSjj33pWTP5tVM8rcSyKJpvz3Snc2SPMKgmtGZled2HwzQemZdE_3yDkzIJntkl4fWpexeNte1sJm0nOMo3d0jAnlF5c5MCcbWR-1iAb-S5ngOyOVCLnTg=&uniplatform=NZKPT&language=CHS

9.Xu2020

LINK:https://qikan.cqvip.com/Qikan/Article/ReadIndex?id=1000002627615&info=c4socuTxv6dv756pg1hm1%2b%2bz0%2fEAcAQ8c2MtxEogx5q5ptPdaY2b6w%3d%3d

10.Zhu2018

LINK:https://kns.cnki.net/kcms2/article/abstract?v=yqeyU9EK6jTB03i6mz6qxOr5XSWGDc5MgjHYPXE8TV7niGtBugDcdiXncxao6AqDlRchNozRFfI_zyfbx01zTBiXlVtrcUPDBBMCTGUmEFrWmpYjace3E7IBpHK56oqqfa-5ZKsdFU=&uniplatform=NZKPT&language=CHS

**DETAILED COMMENTS**

**I. Abstract/Introduction**

***Comment 5:*** *Line 31: This is misleading. Please re-word to, "...t**hat administer BBR in conjunction with standard therapy for to support the treatment of UC".*

**Response 5** Thank you for your meticulous and rigorous guidance; we have made the necessary modifications as requested. (line 28-29 on page 2).

***Comment 6:*** *Line 63: keep style of ratio reporting the same...E.g., you transition from "286.3 per 100,000" to "11.3/100,000"2.*

**Response 6:** Thank you for raising these detailed questions; your rigorous academic attitude is a model for us to learn from. We have changed “11.3/100,000” to “11.3 per 100,000”. (line 51 on page 3).

***Comment 7:*** *Line 74: Italicized genus and species names.*

**Response 7**Thank you for your guidance on the details of our manuscript and your professional advice. We have italicized *Coptis chinensis* and *Phellodendron amurense. (line 62 on page 3).

***Comment 8****You must place your current review/meta-analysis in the context of any existing systematic reviews and meta-analysis on similar content (which do exist)—Describe what content those reviews/meta-analysis include and how your meta-analysis is different and contributes to the literature.*

**Response 8**Thank you very much for your patient guidance. Following your professional advice, we have added a similar meta-analysis study to the manuscript. The details are as follows:

A meta-analysis of animal studies suggests that BBR reduces the disease activity index (DAI) scores and histological colitis score (HCS) in UC animal models[11]. Mechanistically, berberine may exert its effects by decreasing myeloperoxidase (MPO) activity and malondialdehyde (MDA) levels, reducing the expression of pro-inflammatory factors such as interleukin-1β (IL-1β), interleukin 6 (IL-6), and tumor necrosis factor α (TNF-α), and increasing levels of the tight junction proteins zonula occludens-1 (ZO-1) and occludin. However, this animal study evaluates the efficacy of BBR, which does not directly guide its clinical application. As a commonly used adjunctive medication, the effectiveness of BBR combined with 5-ASA treatment for UC is still uncertain, and there is currently a lack of corresponding clinical research. (line 66-75 on page3-4).

***Comment 9*** *Lines 82-84 “The Purpose”: This is misleading; please clarify the purpose to reflect what the meta-analysis is truly evaluating, which is the efficacy of BBR as a complementary therapy to support treatment.*

**Response 9** Thank you for your guidance, we have revised it to the following sentence.

“Therefore, the efficacy of berberine combined with 5-ASA in treating UCremains uncertain. This study aims to evaluate the effects of berberine as an adjunct treatment on the efficacy and safety of UC.” (line 78-79 on page 4).

**II. Methods**

**1.	Search strategy:**

***Comment 10****Just semantics here…but PubMed is technically not a “database”, it is a platform for the database Medline.*

**Response 10**Thank you for your meticulous guidance; we have made subtle adjustments in the manuscript. (line 87 on page 4).

***Comment 11****Were there any limits or filters used? Were there any programs used to convert search syntax across databases (for example, Polyglot)?*

**Response 11** Thank you for your comment. We used specific keywords to search the literature, including "ulcerative colitis," "ulcerative," "berberine," "berberine hydrochloride," "umbellate," "inflammatory bowel disease," "randomized," and "randomized controlled trial". Then, we screened the literature based on the inclusion and exclusion criteria.

Regarding the latter issue, we apologize for the miscommunication, which made it difficult for you to understand. We did not use any program to convert the search syntax across databases. This statement was unnecessary in the text and could lead to misunderstandings, so we have removed it. Lastly, we also thank you for your careful guidance and comments.

**2. *Inclusion criteria***

***Comment 12*** *The second sentence of this section begins “The* *exclusion criteria…” Although, this section is supposed to be about inclusion criteria. Reading this section makes it seem as if you excluded RCTs and studies where BBR was used in conjunction with a certain standard therapy – however, I believe you mean for these to be inclusion criteria.*

**Response 12**e were sorry for our careless mistakes. Thank you for your reminder, we have corrected the “exclusion criteria” into “inclusion criteria”. (line 103 on page 5).

***3. Exclusion Criteria***

***Comment 13*** *“Statistical inaccuracies”: Elaborate and define what you consider inaccuracies, and include how these inaccuracies were identified.*

**Response 13** Thank you very much for your detailed review of our research. We hope that the following explanation can answer your questions.

During the literatre screening process, we found that some studies did not provide specific details such as sex, age, and duration of disease among the basic data. They merely mentioned the basic data of patients in the experimental and treatment groups, stating that after statistical analysis, P > 0.05, indicating that the two groups were comparable. Moreover, specific statistical methods used in the research were not mentioned, and the data results obtained lacked rigorous statistical support. After discussion, we believe that these studies have issues with statistical inaccuracies.

To enhance the persuasiveness of our research, we therefore decided to exclude such similar studies. We hope the above answer can resolve your doubts, and we thank you again for your insightful question. (line 111 on page 5).

***Comment 14*** *“Inadequate original data”: Elaborate and define what you consider inadequate original data, and include how this was identified.*

**Response 14**Thank you for your comment, we think this is a very good question.

Regarding the issue of inadequate original data, we hold the following views. During the literature screening process, we noticed that some studies had very small sample sizes, for example, some studies only involved a few patients per group. Additionally, some studies were lacking supportive data for important indicators such as the Clinical efficacy rate and DAI, with these studies merely mentioning that these indicators improved after treatment. Some studies also failed to mention the specific sources of the original data and their collection methods. In light of these issues, we believe these studies suffer from a lack of adequate original data. (line 111 on page 5).

**4. Secondary outcome measures**

***Comment 15*** *There is a lot of information in the Results section for each measure that belongs here, in the methods.*

**Response 15**Thank you for your professional comment and suggestion. We have condensed and revised the content of this section as shown below:

(1) Baron score: Evaluates the intestinal mucosal damage in patients with active UC. The higher the Baron score, the more severe the colonic mucosal damage.

(2)DAI: The average score of rectal bleeding, stool frequency, and percentage weight change.A higher DAI score indicates more severe UC.

(3)Symptom remission status：The duration over which patients experienced relief from abdominal pain, diarrhea, and pus-involving bloody stools.

(4) Inflammatory cytokines:IL-6，IL-8, TNF-α,IL-10. Levels of IL-6, IL-8, and TNF-α decrease, while IL-10 increases, it indicates a reduction in the inflammatory response of UC.

(5) Immune response: CD4+ T cells,CD8+ T cells ,CD4+/CD8+ ratio. CD4+ and CD8+ T lymphocytes are distinct subpopulations that play pivotal roles in the immune response, with the CD4+/CD8+ ratio serving as a gauge of immune function in UC patients.

(6) Adverse reaction.

(line 133-146 on page 6).

***Comment 16*** *This section would benefit from more clearly defining each measure, what it is used for, and why. Perhaps consider breaking this down into the “three domains” you highlight in the discussion?*

**Response 16**Thank you for your professional guidance. Introducing "three domains" in the results section was an excellent suggestion, and we strongly agree with your perspective. We have made revisions following your request to ensure the results correspond closely with the final discussion, making it easier for readers to understand. (line 119-146 on page 5-6).

***Comment 17*** *Once the reader gets to the discussion, it becomes clear that not all articles contain all measures. In the methods, please define the number of articles used for each outcome.*

**Response 17**Thank you for your reminder, we agree with your point. In the results section, we have defined the number of articles used for each outcome and annotated the references corresponding to each indicator. This will make it easy for the authors to understand which studies specifically mention a certain research indicator, avoiding misunderstandings. The format of our annotations is as follows: “6 studies [24, 26, 28, 30, 32, 33] assessing the serum IL-6 concentration”, “7 studies [24, 26, 28, 30-33] reported on IL-8 levels”. Finally, thank you once again for your help and guidance.

**5. Literature screening, data extraction, and quality assessment**

***Comment 18*** *Were there any auto mation tools or other programs used in the extraction and screening process?*

**Response 18** Thank you for your comment. We apologize for not making the content description clear previously. We used a manual extraction method. We extract the literature information with standardized instructions. In detail as follows. By screening the titles and abstracts, two authors (Jilei Li and Chenchen Zhang) independently removed duplicate and irrelevant records. Any discrepancies between the researchers were addressed by consulting the third researcher Yanchao Xu. (line 150-157 on page 7).

***Comment 19****Were any extraction forms used pretested? (for example, if you created or used an Excel template, were these pretested?*

**Response 19**Thank you for your comment. We did not conduct a pre-test. Instead, we performed a comprehensive extraction of data related to the study and described it in a table. The table's content includes the first author, publication year, sample size, age, sex ratio, interventions, treatment method, treatment time, outcome, and source.

***Comment 20****Was extraction conducted with* *standardized instructions?*

**Response 20**Thank you for your comment. We apologize for not making the content description clear previously. We extract the literature information with standardized instructions. In detail as follows. By screening the titles and abstracts, two authors (Jilei Li and Chenchen Zhang) independently removed duplicate and irrelevant records. Any discrepancies between the researchers were addressed by consulting the third researcher Yanchao Xu. (line 150-155 on page 7).

***Comment 21*** *Were there specific domains for extraction used? If so, what were they?*

**Response 21** Thank you for your comment. Yes, we used specific domains for extraction. Data extraction includes the first author, publication year, sample size, age, sex ratio, interventions, treatment method, treatment time, outcome, and source. (line 155-157 on page 7).

***Comment 22*** *What instruments did you use for risk of bias assessment (Example: ment)?*

**Response 22**Thank you for your comment. We used version 2.0 of the Cochrane Risk-of-Bias (RoB) instrument for risk-of-bias assessment. (line 160-161 on page 7).

***Comment 23*** *How did you handle missing data?*

**Response 23** Thank you for your comment and question. If too much data is missing, it will affect the stability of the results; therefore, we will not include this study. (line 157 on page 7).

**III.	Results**

**1.	Clinical efficacy rate:**

***Comment 24****Lines 204-205: This is the first place where it becomes clear that you are investigating the efficacy of BBR as a complementary therapy, not as a therapy on its own. This needs to be clarified in the introduction and “purpose” of the study.*

**Response 24**

Thank you for your guidance. We have made modifications to the manuscript to avoid creating reading obstacles for the readers. (line 1 on page 1), (line 41-42 on page 2), (line 247 on page 12), (line 409-410 on page 18) et al.

**2.	Baron Score:**

***Comment 25:****Lines 208-213: this definition belongs in the Methods section*

**Response 25:**hank you for your guidance. We have moved this section to the methods section and made some minor modifications. (line 135-136 on page 6).

**3.	DAI score:**

***Comment 26*** *Lines 221-224: this definition belongs in the Methods section*

**Response 26** Thank you for your guidance, We have moved this section to the methods section and made some minor modifications. (line 137-138 on page 6).

**4.	Symptom amelioration analysis:**

***Comment 27*** *Lines 231-233: This information does not belong in the results section. It belongs in either the introduction or the methods section when you introduce/define the biomarkers being measured.*

**Response 27:**Thank you for your guidanc*e. We have transferred this passage to the methods section and made some minor modifications. (line 139-140 on page 6).*

**5.	Inflammatory biomarkers:**

***Comment 28*** *Lines 246-250: This information does not belong in the results section. It belongs in either the introduction or the methods section when you introduce/define the biomarkers being measured.*

**Response 28** Thank you for your comments. Based on your suggestions, we have placed some information in the methods section and have made some minor modifications to ensure smoother language. (line 141-142 on page 6).

**6.	Immune Response6:**

***Comment 29*** *Lines 270-271: regarding the CD4+:CD8+ T cell ratio as a gauge of immune function; this must be cited with a reference.*

**Response 29:**Thank you for your comments, we have added a reference to support this viewpoint, as follows. Smids C, Horjus T H C, Drylewicz J, et al. Intestinal T Cell Profiling in Inflammatory Bowel Disease: LINKing T Cell Subsets to Disease Activity and Disease Course[J]. J Crohns Colitis, 2018,12(4):465-475. (line 143-145 on page 6).

***Comment 30****Line 273: “**Increase in CD4+ T cells” .*

**Response 30**hank you for your guidance. We have not expressed ourselves clearly before, but with your help, we can now articulate more precisely, as follows: "The increase in CD4+ T cells may promote an increase in the number of regulatory T cells, which helps alleviate the inflammatory response in UC." Additionally, we have made modifications to the manuscript accordingly. (line 278-280 on page 14).

***Comment 31*** *I cannot access the articles cited, so I cannot check this: Did these studies specify which CD4+ T cells compartment they measured? Is these T effector cells only, or all CD4+ T cells which include T regulatory cells? This is important to further contextualize the physiological relevance of this finding. CD4+ T cells encompass both T effector cells (e.g., Th1, Th2, Th17, et) AND T regulatory cells, which are immunosuppressive.*

**Response 31**Thank you for your detailed comments and professional guidance. As you pointed out, CD4+ T cells include both T effector cells (such as Th1, Th2, Th17, etc.) and T regulatory cells, which are immunosuppressive. They have different physiological functions; for instance, an increase in Th17 cells may actually indicate the progression of UC, while an increase in Treg cells may suggest a mitigation of UC. We revisited the article and realized that the mentioned CD4+ T cell compartment includes both T effector cells and T regulatory cells. We also provide the DOI or a LINK to the article for easy access by readers.

**IV. Discussion**

**1. General:**

***Comment 32 a.*** *It is a little unclear throughout the Discussion which statements are regarding results from your meta-analysis and which are from other studies.*

**Response 32** We apologize for any confusion caused by unclear writing in our manuscript. We have revised this section and separated our research from other studies for clearer understanding. (line 317 on page 15).

***Comment 33 b.*** *MANY studies use BBR in rodent models of UC that shed light on possible mechanisms of action; It would be useful to place some of your findings in the context of these mechanisms. You very lightly touch on the immunological mechanism, but this can be discussed in more detail.*

**Response 33**Thank you for your professional guidance; below is the information we have added.

Xiong et al. demonstrated through mouse experiments that BBR can improve intestinal mucosal damage, promote mucosal healing, and alleviate symptoms such as diarrhea and bloody stools. Moreover, BBR facilitates the modulation of immune functions in UC mice by activating the IL-4/STAT6 signaling pathway, inhibiting M1 macrophages, and promoting M2 macrophages. M1 and M2 macrophages can influence the differentiation of CD4+ and CD8+ T cells by producing factors like TNF-α and IL-10, thereby regulating the immune function in UC mice and suppressing excessive inflammatory responses. Li et al.research suggests that BBR can alleviate the inflammatory response in a cat model of UC, reduce inflammatory factors, and improve conditions of diarrhea and bloody stools. Li et al. further mechanistic studies showed that BBR can decrease Bacteroidetes and increase Firmicutes in DSS-induced cats. BBR reshaped the microbiota composition by reducing the abundance of Proteobacteria. (line 322-333 on page 15).

***Comment 34*** *BBR also clearly interacts with the gut microbiome to modulate immune function and intestinal health. However, this important interaction is not mentioned at all. B*

**Response 34** Thank you for your comment. We have revisited the studies included in our research and did not find any that addressed issues related to gut microbiota. The focus of our paper is to explore the effects of berberine as an adjunctive treatment for UC, and we did not investigate the mechanisms of its therapeutic action. However, in the discussion section, we touched upon some basic research on how berberine treats UC by modulating the gut microbiome. Your suggestion is very meaningful and has provoked further thought; we will explore this aspect in future research. (line 331-333 on page 16).

***Comment 35*** *Breaking down your outcomes into these “three domains” is a great way to organize the information for the reader. Perhaps consider introducing “domains” of outcomes earlier on in the manuscript.*

**Response 35**Thank you for your professional and meticulous comments; we highly agree with your suggestions. According to your guidance, we have introduced the 'three domains' of results in the Outcome Measures section. Below are the specific statements.

Primary outcome measures—Symptomatic relief

（1）The clinical efficacy rate

……

Secondary outcome measures—Serological indicators and Safety profiles

(1) Baron score: Evaluates the intestinal mucosal damage in patients with active UC[17]. The higher the Baron score, the more severe the colonic mucosal damage.

(2)DAI: The average score of rectal bleeding, stool frequency, and percentage weight change.A higher DAI score indicates more severe UC

(3)Symptom remission status：The duration over which patients experienced relief from abdominal pain, diarrhea, and pus-involving bloody stools.

(4) Inflammatory cytokines:IL-6，IL-8, TNF-α,IL-10. Levels of IL-6, IL-8, and TNF-α decrease, while IL-10 increases, it indicates a reduction in the inflammatory response of UC.

(5) Immune response: CD4+ T cells,CD8+ T cells ,CD4+/CD8+ ratio. CD4+ and CD8+ T lymphocytes are distinct subpopulations that play pivotal roles in the immune response, with CD4+/CD8+ ratio serving as a gauge of immune function in UC patients.

(6) Adverse reaction.

(line 119-146 on page 5-6).

***Comment 36*** *Line 320: “casting doubt on robustness..”*

*I would steer away from the term "casting doubt" and focus more on facts... limited sample sizes can make studies underpowered and, if homogeneous samples were used (i.e., geographically, or demographically), results may not be generalized to the wider population.*

**Response 36**Thank you for your professional comments and suggestions; your guidance has been of great assistance to us. We have made modifications to this section according to your advice. (line 403-407 on page 18).

***Comment 37*** *Lines 331-334: this sentence is a little hard to follow*

**Response 37** Due to our expression issues, we apologize for the difficulties caused in your reading and appreciate your guidance. To help readers understand better, we have revised the sentence to the following: "Furthermore, BBR can reduce the DAI scores and shorten the duration of symptoms such as diarrhea, rectal bleeding, and abdominal pain, indicating that BBR as a supplementary treatment has a certain efficacy in managing UC." (line 354-356 on page 16).

***Comment 38*** *In the following sentence, you mention BBR lowering Baron scores and that lowering of score is correlated to symptom relief. However, here in line 335 you mention "surge" in Baron score, which indicates an elevation in score. These two statements are contradictory.*

**Response 38**Thanks for your careful checks. We are sorry for our carelessness. Following your suggestion, we have changed "surge" to "reduction" to ensure the sentence remains consistent in meaning throughout. (line 357 on page 16).

***Comment 39	Limitations:*** *There are limitations to this study which must be highlighted in the discussion.*

**Response 39** Thank you for your comments, and we also appreciate the valuable suggestions you have made. We have added content under "Limitations" as follows.

However, there are certain limitations in this study. For example, all articles included in the meta-analysis originate from China/Asia. This limits the generalizability of the results. Additionally, there is variability in the administration routes and treatment duration across different studies, which may limit the application of berberine as an adjunctive treatment. Future research should focus on conducting more rigorous, large-sample, multi-center clinical trials for further validation. (line 402-406 on page 18).

***Comment 40****.*	*All articles included in the meta-analysis are from China/Asia. This limits the generalizability of the results.*

***Response 40***Thank you for your comments and guidance; we have supplemented our document with the content you mentioned. (line 402-404 on page 18).

***Comment 41:****able 1 highlights differences in administration route and treatment time. Could these factors be confounders to analysis?*

**Response 41:**Yes, thank you for your comments and guidance; we have supplemented our document with the content you mentioned. (line 404-406 on page 18).

***Comment 42 :****verstating findings: You are not evaluating the efficacy of BBR as an alternative therapy in this meta-analysis, you are evaluating BBR as a complementary therapy. Thus, your meta-analysis supports the concept of “BBR as a safe and efficacious complementary intervention to support disease management”. Specific lines in manuscript that overstate findings/context of article:*

**Response 42:**hank you for your professional and careful comments; we fully agree with your thoughts. Our previous expression was not clear enough, and it overstated our research findings. We have made revisions, using sentences like "treatment of UC with berberine combined with 5-ASA" or "BBR as a safe and efficacious complementary intervention to support disease management", to allow readers to better understand our study.

***Comment 43****Line 309-310: “BBR has emerged as a promising alternative therapy for treating this disease”*

**Response 43**Thank you for your comment; we have revised the sentence from "BBR has emerged as a promising alternative therapy for treating this disease" to " BBR can serve as a safe and efficacious complementary intervention to support UC disease management." (line 320 on page 15).

***Comment 44***	*Line 312: “…application of BBR in treating UC…”*

**Response 44** Thank you for your guidance. We have changed "...application of BBR in treating UC..." into "...application of BBR plus 5-ASA in treating UC..." (line 334 on page 16).

***Comment 45***	*Line 371: “BBR as a safe and efficacious intervention”*

**Response 45** Thank you for your guidance. We have revised "BBR as a safe and efficacious intervention" into "The research results reported in this article suggest that BBR plus 5-ASA for UC is a safe and effective intervention." (line 410 on page 18).

**4.	Discussion of immune responses:**

***Comment 46*** *Lines 340-342: regarding IL-8 secretion; this must be cited with a reference.*

**Response 46**Thank you for your guidance. We have added the reference below here.

[44]Salah N, Dubuquoy L, Carpentier R, Betbeder D. Starch nanoparticles improve curcumin-induced production of anti-inflammatory cytokines in intestinal epithelial cells. Int J Pharm X. 2022 Mar 8;4:100114. doi: 10.1016/j.ijpx.2022.100114. PMID: 35295898; PMCID: PMC8919232.(line 367 on page 17).

***Comment 47*** *Lines 342-344: Regarding BBR effect on NFkB and cytokines; this must be cited with a reference*

**Response 47**Thank you for your guidance. We have added the reference below here.

[45]Laurindo LF, Santos AROD, Carvalho ACA, Bechara MD, Guiguer EL, Goulart RA, Vargas Sinatora R, Araújo AC, Barbalho SM. Phytochemicals and Regulation of NF-kB in Inflammatory Bowel Diseases: An Overview of In Vitro and In Vivo Effects. Metabolites. 2023 Jan 7;13(1):96. doi: 10.3390/metabo13010096. PMID: 36677021; PMCID: PMC9862976.

(line 369 on page 17) .

***Comment 48*** *Lines 351-352: The wording of this is not entirely correct and is a little misleading. An inflammatory insult does not cause de novo CD4+ and CD8+ cell "synthesis" (any "synthesis" would happen in the thymus)... I think what you are trying to convey here is that naive CD4+ T cells and naive CD8+ T cells become activated and differentiate into effector cells, memory cells, etc. Regarding the use of the word "evolution"… you should really be referring to this as the “activation and differentiation" of naive CD4+ T cells.*

**Response 48**Thank you for your guidance, we fully agree with your views. We have made the changes as per your request. (line 376-379 on page 17).

***Comment 49*** *:nes 354-355: Information cited with Reference 35; this is not the best reference for this statement. Please double-check.*

**Response 49:**hank you for your guidance on the details of the article. We were really sorry for our careless mistakes. We have changed the previous reference to the following one.

[48]Zebley CC, Gottschalk S, Youngblood B. Rewriting History: Epigenetic Reprogramming of CD8+ T cells Differentiation to Enhance Immunotherapy. Trends Immunol. 2020 Aug;41(8):665-675. doi: 10.1016/j.it.2020.06.008. Epub 2020 Jul 2. PMID: 32624330; PMCID: PMC7395868.

(line 379 on page 17).

***Comment 50:****ines 355-357: Regarding the ratio of CD4+:CD8+ T cells and which ratio is indicative of immune competency; this must be cited with a reference.*

**Response 5****0:**ank you for your guidance. We have added the reference below here.

[51]Bolivar-Wagers S, Larson J H, Jin S, et al. Cytolytic CD4(+) and CD8(+) Regulatory T-Cells and Implications for Developing Immunotherapies to Combat Graft-Versus-Host Disease[J]. Front Immunol, 2022,13:864748.

(line 385 on page 17).

***Comment 51*** *Also, it is a little unclear what the “range” of 1 to 2 means. Is this the range of ratios?*

**Response 51**Yes, this is the range for the CD4+/CD8+ T cells ratio.To better help readers understand, we have changed the previous sentence to “Under normal circumstances, the CD4+/CD8+ ratio falls between 1 and 2, indicating healthy immune function. An imbalance in the CD4+/CD8+ ratio could exacerbate the inflammatory condition in patients with UC”. Additionally, we have also added references here. (line 383-385 on page 17).

***Comment 52****Line 358: “…enhancing immunological functions.”This statement is very general, and its meaning is unclear. Please clarify what you mean by "enhancing." Is the immunological tone of intestinal mucosa becoming more activated and inflammatory? Is it becoming more suppressed?*

**Response 52**Thank you for your feedback. Regarding the term "enhancing immunological functions".We refer to the capability of BBR to modulate CD4+ and CD8+ T cells and their ratio, thereby promoting immune balance and enhanced function. The enhancement of immune functions not only includes the strengthening of immune responses in areas such as the gut mucosa, making it more effective in defending against pathogens but also in mitigating excessive inflammatory responses, thereby preventing damage to tissues. This, in turn, can alleviate the suffering of patients with UC. Hence, BBR's positive influence on the immune system is manifested both in enhancing immune activity and in controlling inflammation, thereby achieving a more balanced and efficient immune state. (line 385 on page 18).

**V. Other:**

***Comment 53:****Citation 22: Pengli W. Salt of berberine combined with mesalazine for the treatment of ulcerative colitis: an observational study on the therapeutic effects[J]. Clinical Medical Engineering, 2020,27(6):771-772.a. Issue 1: Is this the “Wang 2020” article? If so, please correct the citation.* *b. Issue 2: There is concern over the title which mentions, “observational study” where the studies included in the meta-analysis should be randomized controlled trials. However, I cannot find this study through searches of Medline, Embase, or my institutional libraries to check this study.*

**Response 53:**Thank you for your comment. We are sorry for our carelessness. This refers to the "Wang 2020" article, which, upon our re-verification, indeed falls under the category of randomized controlled trials. We have re-translated the title of the study and cited the article in the following format.

[29]Wang P. Salt of berberine combined with mesalazine for the treatment of ulcerative colitis: an observational study on the therapeutic effects[J]. Clinical Medical Engineering, 2020,27(6):771-772.

***Comment 54:****itation 24: Liuliu W. The therapeutic efficacy of berberine combined with mesalazine and its impact on inflammatory factors in patients with distal ulcerative colitis[J]. Proceeding of Clinical Medicine, 2019,28(7):506-508.*

*Issue 1: Is this the “Wei 2019” article? If so, please correct the citation.*

**Response 54**Thank you for your comment. We are sorry for our carelessness. Yes， We have already corrected the citation. The specifics are as follows:

[31]Wei L. The therapeutic efficacy of berberine combined with mesalazine and its impact on inflammatory factors in patients with distal ulcerative colitis[J]. Proceeding of Clinical Medicine, 2019,28(7):506-508.

***Comment 55****Concerns over citations: Lack of in-text citation for many statements.*

**Response 55:**Thank you for your guidance. We have overlooked this issue before, and we appreciate you pointing out our mistake promptly. It was only with your help that we quickly realized the necessity of proper referencing. Following your valuable advice, we have added references and citations accordingly. We will keep this in mind and make sure similar errors do not occur in the future. In conclusion, we are very grateful for your assistance.

***Comment 56:****•	Articles listed in Table 1 must be cited with the citation number of the article (as numbered in the references section) so the full citations can be easily found.*

**Response 56:**

Thank you for your guidance, we have listed the citation numbers of the articles in Table 1.

(line 187-190 on page 9-10).

***Comment 57:***	*Line 80 “Xu” article: check in-text citation style. Should a date be included?*

**Response 57:**Thank you for your guidance. We have modified the citation style to "Xu 2020".(line 75 on page 4).

***Comment 58:***	*Lines 269-271: This sentence regarding the ratio of CD4+:CD8+ being used as an indicator of immune function must be cited with a reference.*

**Response 58:**

Thank you for your guidance; below are the referenced citations.

[22]Smids C, Horjus Talabur Horje CS, Drylewicz J, Roosenboom B, Groenen MJM, van Koolwijk E, van Lochem EG, Wahab PJ. Intestinal T Cell Profiling in Inflammatory Bowel Disease: LINKing T Cell Subsets to Disease Activity and Disease Course. J Crohns Colitis. 2018 Mar 28;12(4):465-475. doi: 10.1093/ecco-jcc/jjx160. PMID: 29211912. (line 145 on page 6).

***Comment 59:***	*Lines 308-309: This sentence must be cited with a reference.*

**Response 59:**Thank you for your guidance; below are the referenced citations.

[35] Feuerstein J D, Moss A C, Farraye F A. Ulcerative Colitis[J]. Mayo Clin Proc, 2019,94(7):1357-1373.

(line 319 on page 15).

***Comment 60:***	*Lines 322-325: Cite which references this applies to.*

**Response 60:**First of all, thank you very much for your thoughtful comments. Your academic attitude is a role model for us to follow. We have reanalyzed the discussion section and found that this sentence is unnecessary in the article and can easily lead to misunderstanding. To make the paragraph more fluent and easier to understand, we have deleted this sentence. Thank you once again for your guidance.

***Comment 61:***	*Lines 326-327: The sentence regarding remission and relapse must be cited with a reference.*

**Response 61:** Thank you for your guidance; below are the referenced citations.

[41]Neurath MF, Vieth M. Different levels of healing in inflammatory bowel diseases: mucosal, histological, transmural, barrier and complete healing. Gut. 2023 Nov;72(11):2164-2183. doi: 10.1136/gutjnl-2023-329964. Epub 2023 Aug 27. PMID: 37640443. (line 344 on page 16).

***Comment 62:***	*Lines 329-330: The sentence regarding endoscopic assessments must be cited with a reference. It is also unclear if this is from your meta-analysis or a different study.*

**Response 62:**We apologize, this was our mistake. Our unclear expression caused you difficulties in reading. This sentence stems from our research, so it does not require a citation. To ensure a better understanding for the reader, we have revised the sentence to read as follows: "The results of the endoscopic assessments in our study suggest that following BBR intervention, BBR is instrumental in lowering Baron scores, hence fostering mucosal restitution." (line 352-354 on page 16).

**Reviewer #2:**

***Comment 1:*** *Authors should provide more detailed information on the sources of BBR in each study.*

**Response 1:** Thank you for your comment; we have summarized the information on the sources of Berberine. Due to the extensive content, including it in Table 1 would disrupt the overall format of the table, so we have uploaded this part as supplementary material. The specific content is shown in the following table.

***Comment*** *2: It is recommended to include the UC pathological staging of patients before treatment in the study.*

**Response 2:**Thank you for your advice and guidance. Following your suggestions, we have carefully reviewed the articles involved in this meta-analysis. Regrettably, they do not document the UC pathological staging. We believe the likely reason is that UC is classified according to various standards during clinical treatment. For instance, UC can be categorized based on symptom presentation into active stages (characterized primarily by abdominal pain, diarrhea, and bloody purulent stools) or remission stages (with no significant symptoms of abdominal pain, diarrhea, or bloody purulent stools). Additionally, UC can be classified according to the severity of symptoms into mild, moderate, or severe categories. We have extracted this information and summarized it in the table below, as shown in the following table.

***Comment 3:*** *Please calibrate the reference format in the manuscript to maintain*

**Response 3:**Thank you for your comment. We have revised the format of the references to ensure consistency.

***Comment 4:*** *The title is "Efficacy and safety of berberine for ulcerative colitis: A systematic review and meta-analysis", but the majority of the data included in the meta-analysis is related to the combined treatment of BBR and 5-ASA. How to demonstrate the efficacy and safety of BBR through the use of BBR in combination with 5-ASA data? Please use concise and succinct language to describe your research findings regarding this matter.*

**Response 4:**Thank you very much for your detailed review and profound insights into our research. We hope that the following explanation can answer your questions. In the literature included in our study, the control group received standard 5-ASA treatment, while the treatment group received BBR combined with 5-ASA treatment. Research findings showed that adding BBR to the treatment regimen along with 5-ASA could improve the clinical efficacy rate and reduce DAI scores compared to using 5-ASA alone. These results suggest that BBR as an adjunct therapy for UC has certain potential. Finally, we also revised the ambiguous sentences in the manuscript to enhance the readers' understanding. (line 1 on page 1), (line 41-42 on page 2), (line 247 on page 12), (line 409-410 on page 18) et al.
