# Supplementary material for: Efficacy and safety of berberine plus 5-ASA for ulcerative colitis: A systematic review and meta-analysis
Source: PLoS One. 2024 Sep 6;19(9):e0309144. doi: 10.1371/journal.pone.0309144 (PMC11379390; doi:10.1371/journal.pone.0309144)
Supplement: Attachment — Submitted filename: Responses to Reviewers.docx [file pone.0309144.s007.docx]

Dear Editors and Reviewers:

We gratefully appreciate the editors and all reviewers for their time spent making positive and constructive comments. These comments are all valuable and helpful for revising and improving our manuscript entitled “Efficacy and safety of berberine plus 5-ASA for ulcerative colitis: A systematic review and meta-analysis” (PONE-D-24-02154).

We have made our utmost efforts to improve the manuscript in the following ways: (1) Correct grammatical errors in the manuscript;(2) Review the manuscript’s formatting and simplify complex sentences for better readability;(3) Revise the manuscript according to the reviewers' comments, such as changing “effector cells, memory cells” to “effector cells and/or memory cells. ”

These revisions will help enhance the overall quality of the manuscript. The reviewer comments are laid out below in italicized font and specific concerns have been numbered. We have studied comments carefully and have made corrections which we hope meet with approval. Our response is given in normal font and changes/additions to the manuscript are given in red.

Thank you and best regards.

Yours sincerely,

Lili Yang

Kaifeng Central Hospital

**Responses to Reviewers**

***Comment 1:*** *Under the Inclusion Criteria section-- No need to use colons (the “:” symbol) after symptomatic relief, serological indicators, etc., because the lists that follow are encapsulated in parentheses.*

**Response 1:** Thank you for your careful and professional guidance. We have removed the ":" in that section as you suggested. (line 107-109 on page 5)

***Comment 2:*** *Under the Exclusion Criteria section-- Line 117, there is a period where there should be a semi-colon.*

**Response 2:** Thank you for reading our paper so thoroughly. We have made the necessary revisions. (line 115 on page 5)

***Comment 3:*** *In the Discussion, line 379-- Currently “effector cells, memory cells” --> “effector cells AND/OR memory cells.”*

**Response 3:**Thank you for your guidance. We have made the revisions according to your suggestions.

(line 388 on page 18)

***Comment 4****:Character spacing throughout the manuscript -- There are many locations where there is no space between characters that should have spaces (e.g., between a period and a new word, or between a colon and a new word, etc.).*

**Response 4:** Thank you for your rigorous and specific review comments, which will enhance the readability of our manuscript. We also apologize for our errors. After a thorough review, we have corrected the incorrect character spacing in the manuscript. Here are a few examples of the revisions; more changes can be seen in the manuscript:

(1). We have added a space before the reference number in the citations. For example, changed “patients with active UC[17]” to “patients with active UC [17]”. (line 134 on page 6)

(2). Changed “(2)DAI” to “(2) DAI” (line 136 on page 6)

(3). Changed “Inflammatory cytokines: IL-6” to “Inflammatory cytokines: IL-6” (line 140 on page 6)

(4). Changed “BBR: 0.2g,tid” to “BBR: 0.2 g, tid”, “E:33.87±3.42” to “E: 33.87±3.42” (line 187 on page 10)

At the same time, we are also making revisions to other issues. Below are a few examples of the changes, with more modifications detailed in the manuscript.

(1). We have rephrased complex sentences. For instance, “*Of particular interest is BBR, an efficacious alkaloid isolated from botanical sources such as Coptis chinensis and Phellodendron amurense, which was identified for its ability to relieve symptoms of abdominal discomfort and diarrhea within UC treatment paradigms*” **has been changed to** “*BBR, a potent alkaloid from Coptis chinensis and Phellodendron amurense, is noted for easing abdominal discomfort and diarrhea in UC treatment.*” (line 62-63 on page 3)

(2). Standardized symbols: Changed “*For the retrieval of literature, the search terms used were ‘ulcerative colitis’, ‘ulcerative’, ‘inflammatory bowel disease’, ‘BBR’, ‘BBR hydrochloride’, and ‘umbellate’ ‘randomized’ and ‘randomized controlled trial*” **has been changed to** “*For the retrieval of literature, the search terms used were ‘ulcerative colitis’, ‘ulcerative’, ‘inflammatory bowel disease’, ‘BBR’, ‘BBR hydrochloride’, ‘umbellate’, ‘randomized’, and ‘randomized controlled trial’.*” (line 88-91 on page 4)

(3). Changed “in the treatment of UC” to “in treating UC”(line 121 on page 5)

(4). Changed “flow—chart” to “flowchart” (line 178 on page 8)

(5). Changed “w: Week” to “W: Week” (line 188 on page 10)

(6). Changed “I2” to “I^2^” (line 215 on page 11)

(7). Changed “（4）” to “(4) ” (line 256 on page 13)

(8). Changed “those with UC” to “UC patients” (line 353 on page 16)

(9). Changed “our” to “Our” (line 381 on page 17)

(10). Changed “Inflammatory” to “inflammatory” (line 382 on page 17)…

***Comment 5****: Under the Primary Outcome Measures section-- Check the numbering. Both “clinical efficacy rate” and “complete remission” are labeled with the number 1.*

**Response *5****:* Thank you for carefully reading our manuscript and providing valuable guidance. I believe we need explain the following: In the "Under the Primary Outcome Measures" section, (1) The evaluation of "clinical efficacy rate" includes three aspects, namely ①Complete remission; ②Effective treatment; and ③Ineffective treatment. Additionally, we have used different notations for "1" in the text. For example, we used (1) to annotate "clinical efficacy rate" and ① to annotate "complete remission". These two annotations are distinct. Based on the above situation, we have not made any changes here. Thank you again for your guidance. (line 120 on page 5)

***Comment 6:*** *Additionally, while you have figure titles, you still do not have figure legends. Please add these.*

**Response 6:** Thank you for your guidance and comments. We have added the figure legends as requested and marked them in red in the manuscript. The details are as follows.

Fig 1. PRISMA flowchart of the study selection process. A total of 10 studies meeting the requirements were included. (line 178 on page 8)

Fig 2. Risk of bias summary. The quality of the included studies was evaluated. (line 209 on page 11)

Fig 3. Meta-analysis of Clinical effective rate. Berberine adjunctive therapy can improve the clinical effective rate. (line 218 on page 11)

Fig 4. Meta-analysis of Baron score. Berberine adjunctive therapy can reduce the Baron score. (line 229 on page 12)

Fig 5. Meta-analysis of DAI score. Berberine adjunctive therapy can reduce the DAI score. (line 238 on page 12)

Fig 6. Meta-analysis of clinical symptoms. Berberine adjunctive therapy can alleviate symptoms of abdominal pain, diarrhea, and hematochezia. (line 253 on page 13)

Fig 7. Meta-analysis of inflammatory cytokines. Berberine adjunctive therapy can reduce levels of IL-6, IL-8, and TNF-α and increase IL-10 levels. (line 279 on page 13)

Fig 8. Meta-analysis of CD4+ T cells, CD8+ T cells, CD4+/CD8+ ratio. Berberine adjunctive therapy can increase levels of CD4+ T cells and the CD4+/CD8+ T cell ratio, with no significant effect on CD8+ T cell levels. (line 297 on page 14)

Fig 9. Meta-analysis of adverse reactions. Berberine adjunctive therapy has no significant adverse reactions. (line 309 on page 15)

Fig 10. Clinical effective rate funnel chart. The clinical effectiveness rate showed no significant bias risk. (line 324 on page 15)
